# Supplementary material for: Trypsin/Zn3(PO4)2 Hybrid Nanoflowers: Controlled Synthesis and Excellent Performance as an Immobilized Enzyme
Source: Int J Mol Sci. 2022 Oct 6;23(19):11853. doi: 10.3390/ijms231911853 (PMC9569851; doi:10.3390/ijms231911853)
Supplement: Supplementary file 1 [file ijms-23-11853-s001.zip › ijms-1910333-supplementary.pdf]

International Journal of *Molecular Sciences*

MDPI

*Supplementary file*

# Trypsin/$Zn_3(PO_4)_2$ Hybrid Nanoflowers: Controlled Synthesis and Excellent Performance as an Immobilized Enzyme

**Zichao Wang †, Pei Liu *,†, Ziyi Fang and He Jiang**

The Key Laboratory of Space Applied Physics and Chemistry, Ministry of Education, Shaanxi Key Laboratory of Macromolecular Science and Technology, School of Chemistry and Chemical Engineering, Northwestern Polytechnical University, Xi'an 710072, China
* Correspondence: liupei@nwpu.edu.cn
† These authors contributed equally to this work.

**Citation:** Wang, Z.; Liu, P.; Fang, Z.; Jiang, H. Trypsin/$Zn_3(PO_4)_2$ Hybrid Nanoflowers: Controlled Synthesis and Excellent Performance as an Immobilized Enzyme. *Int. J. Mol. Sci.* **2022**, *23*, 11853. https://doi.org/10.3390/ijms231911853

Academic Editor: Antonino Mazzaglia

Received: 26 August 2022
Accepted: 1 October 2022
Published: 6 October 2022

**Publisher's Note:** MDPI stays neutral with regard to jurisdictional claims in published maps and institutional affiliations.

**Copyright:** © 2022 by the authors. Licensee MDPI, Basel, Switzerland. This article is an open access article distributed under the terms and conditions of the Creative Commons Attribution (CC BY) license (https://creativecommons.org/licenses/by/4.0/).

**Table S1.** Data for the synthesis of trypsin/$Zn_3(PO_4)_2$ hybrid nanoflowers.

| No. | Trypsin (g) | $Zn(Ac)_2·2H_2O$ (g) | Reaction time (min) |
|---|---|---|---|
| 1 | 0 | 0.56 | 120 |
| 2 | 0.01 | 0.56 | 120 |
| 3 | 0.0250 | 0.56 | 120 |
| 4 | 0.05 | 0.56 | 120 |
| 5 | 0.10 | 0.56 | 120 |
| 6 | 0.25 | 0.56 | 120 |
| 7 | 0.05 | 0.42 | 120 |
| 8 | 0.05 | 0.28 | 120 |
| 9 | 0.05 | 0.14 | 120 |
| 10 | 0.0250 | 0.56 | 10 |
| 11 | 0.0250 | 0.56 | 20 |
| 12 | 0.0250 | 0.56 | 30 |
| 13 | 0.0250 | 0.56 | 60 |
| 14 | 0.30 | 0.56 | 120 |

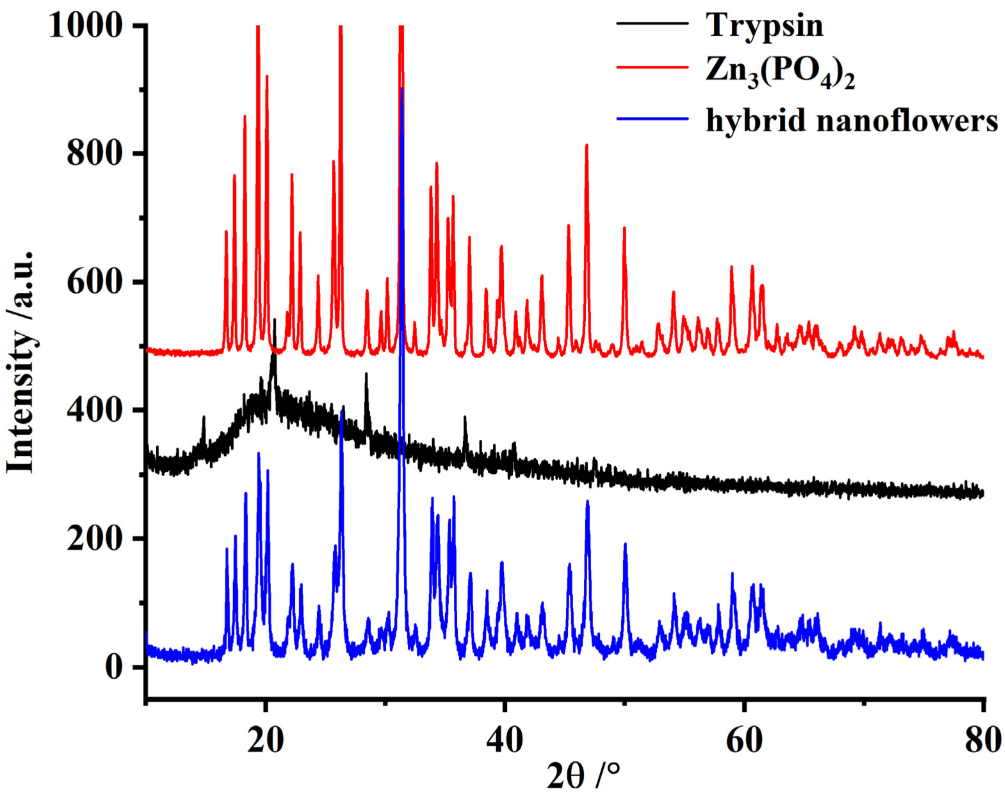


**Figure S1.** XRD patterns of the trypsin, $Zn_3(PO_4)_2$ and trypsin/$Zn_3(PO_4)_2$ hybrid nanoflowers.

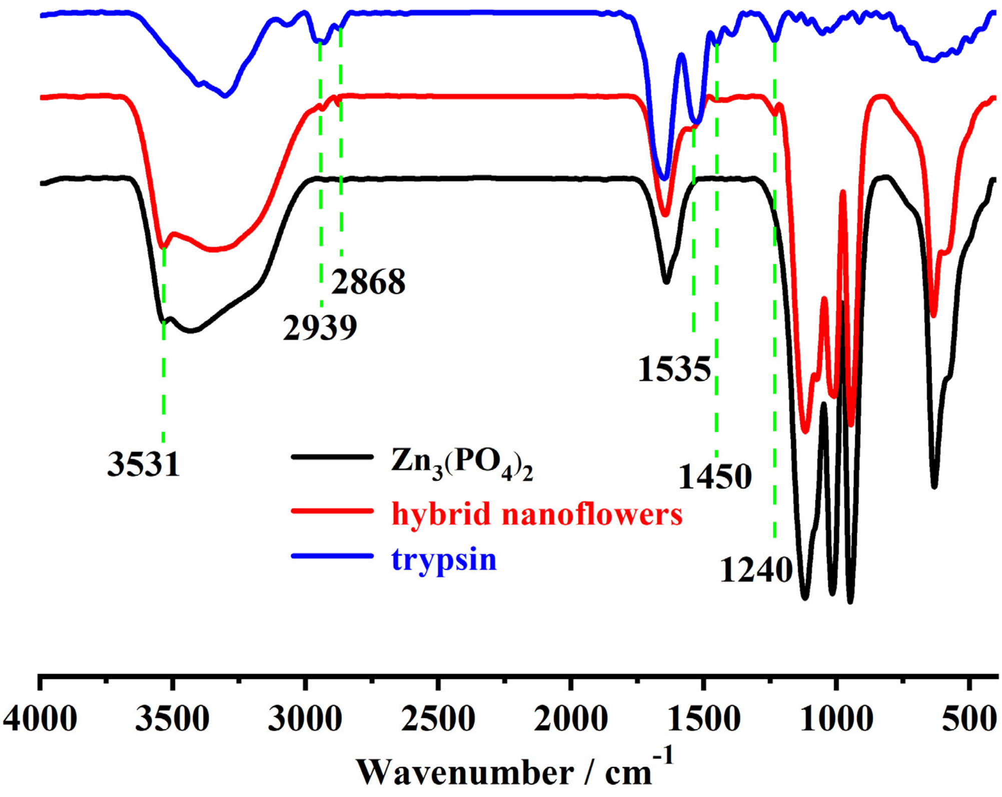


**Figure S2.** FTIR spectra of the trypsin, $Zn_3(PO_4)_2$ and trypsin/$Zn_3(PO_4)_2$ hybrid nanoflowers.

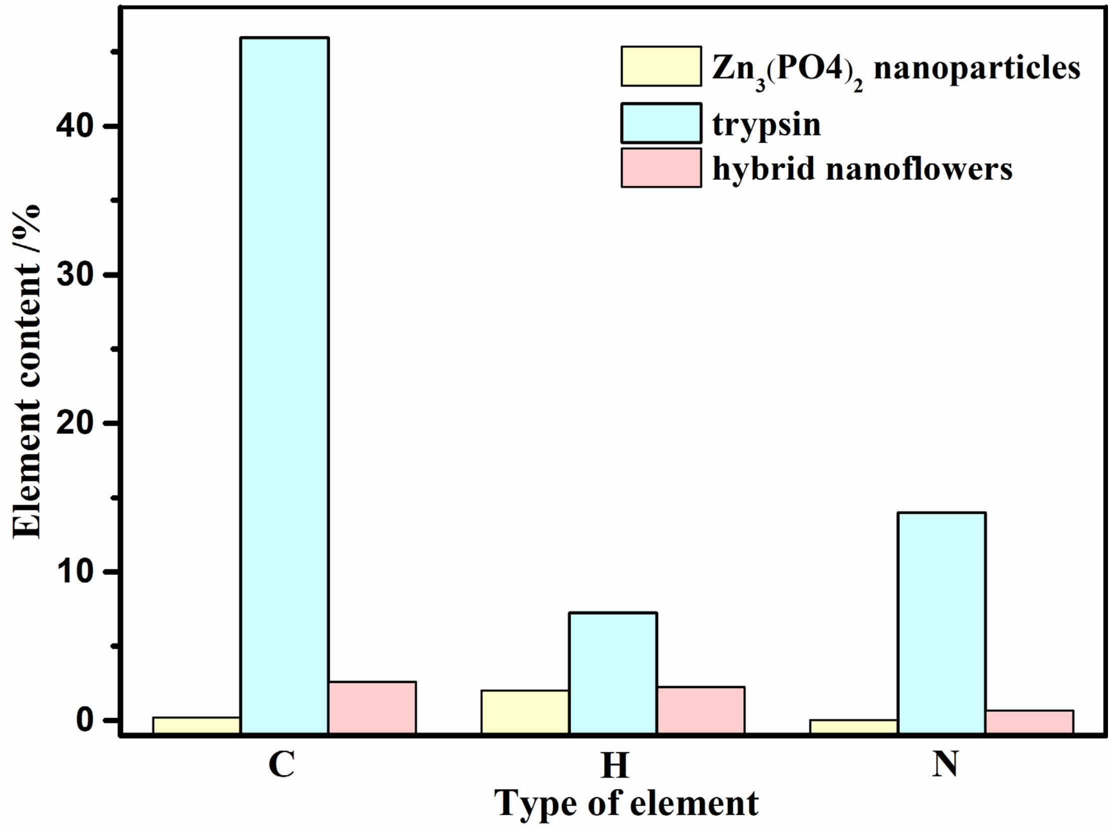


**Figure S3.** Elemental analysis of the trypsin, $Zn_3(PO_4)_2$ and trypsin/$Zn_3(PO_4)_2$ hybrid nanoflowers.

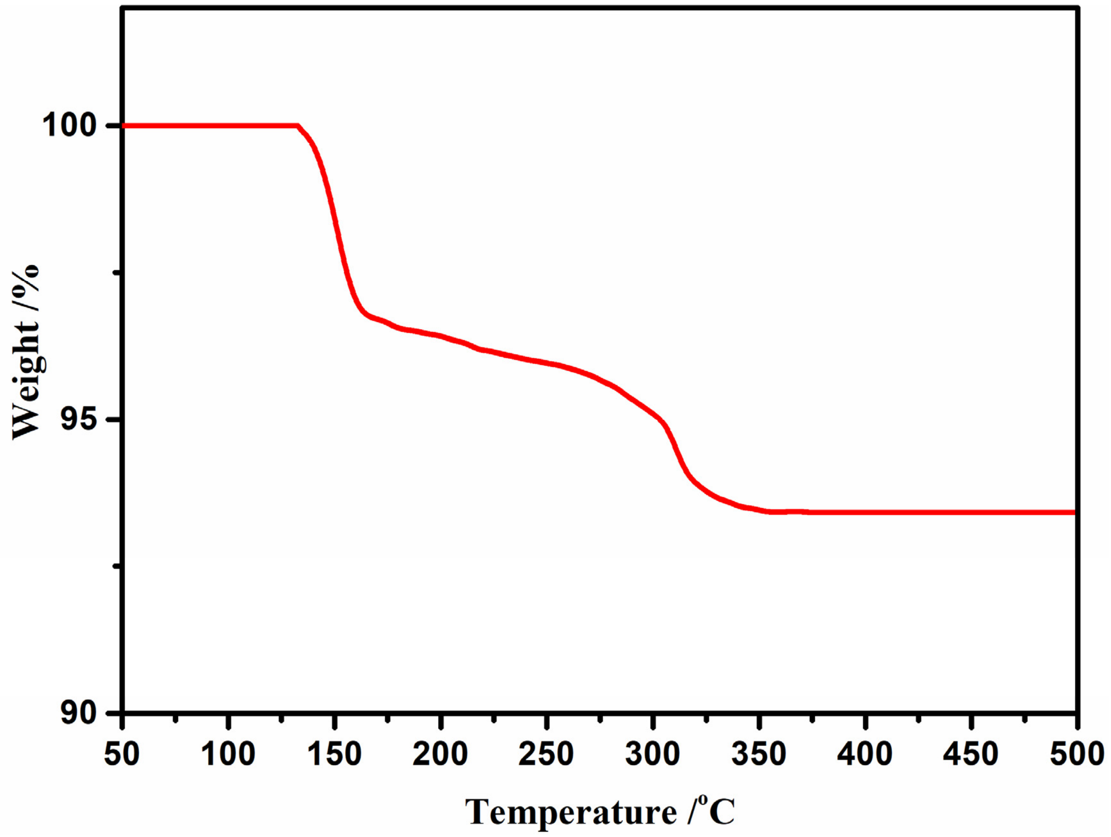


**Figure S4.** TGA curve of trypsin/$Zn_3(PO_4)_2$ hybrid nanoflowers.

**Table S2.** Date for specific surface area and average pore diameter of trypsin/$Zn_3(PO_4)_2$ hybrid nanoflowers with different amount of trypsin.

| Addition amount of trypsin (g) | specific surface area ($m^2/g$) | average pore diameter (nm) |
|---|---|---|
| 0 | 38.68 | 13.28 |
| 0.01 | 32.55 | 19.26 |
| 0.025 | 35.33 | 16.28 |
| 0.05 | 42.96 | 13.04 |
| 0.1 | 56.67 | 11.95 |
| 0.25 | 68.35 | 11.20 |

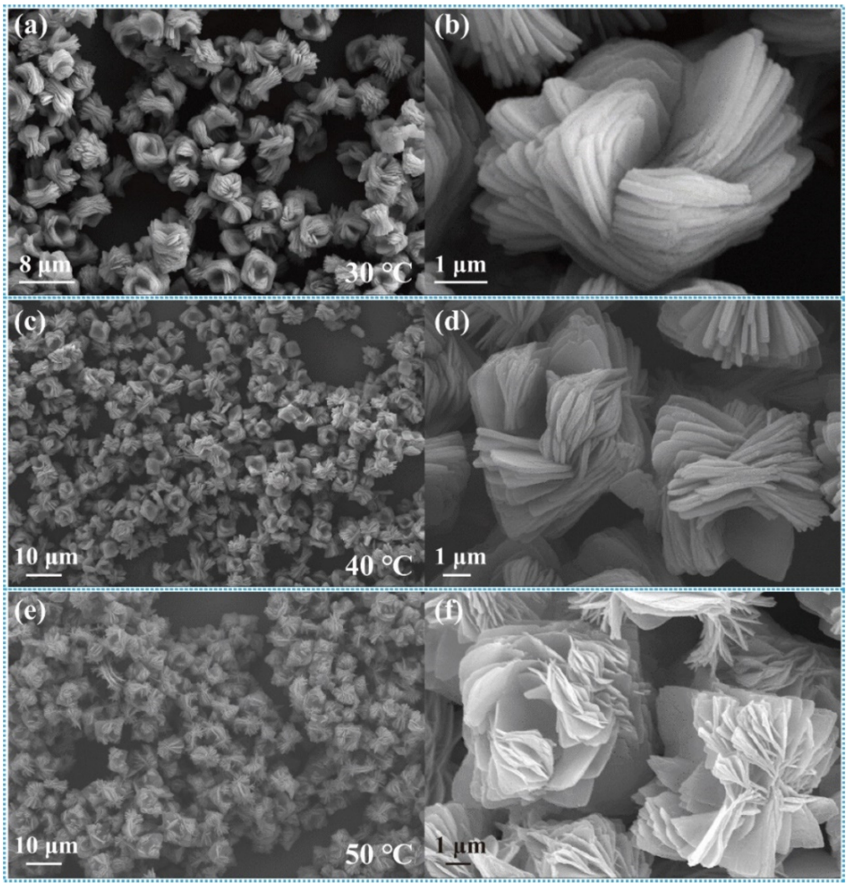


**Figure S5.** The SEM images morphology of trypsin/$Zn_3(PO_4)_2$ hybrid nanoflowers prepared at (a, b) 30 °C, (c, d) 40 °C, (e, f) 50 °C.

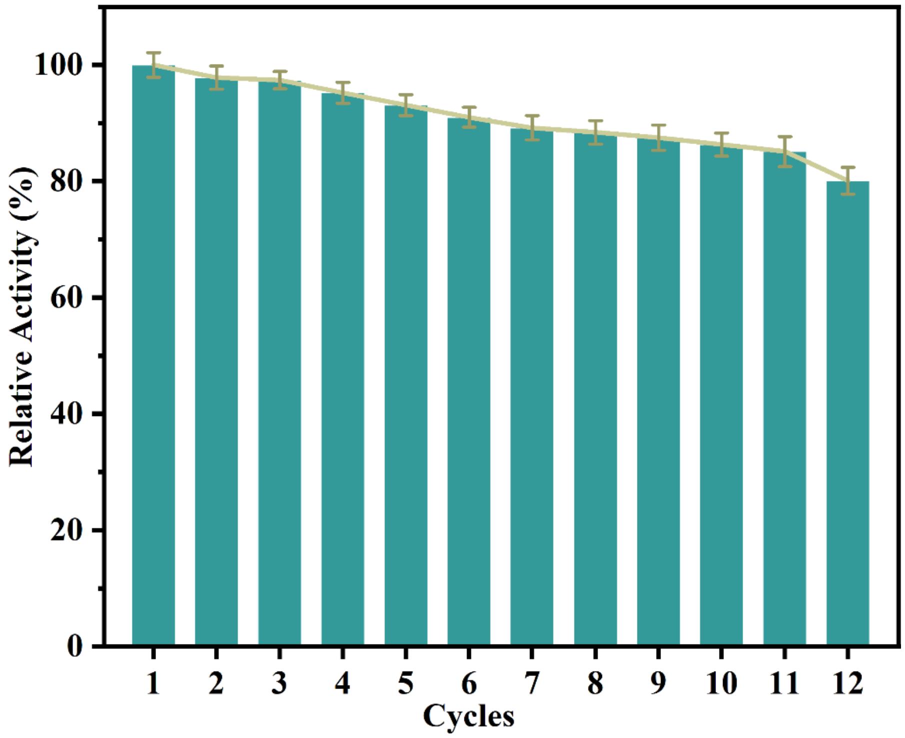


**Figure S6.** Reusability of trypsin/$Zn_3(PO_4)_2$ hybrid nanoflowers.
